# Pulmonary Manifestations of Birt–Hogg–Dubé Syndrome: A Single-Centre Retrospective Case Series of Seven Genetically Confirmed Patients

**DOI:** 10.3390/jcm15155820

**Published:** 2026-07-25

**Authors:** Anna Annunziata, Lidia Atripaldi, Roberto Rega, Anna Michela Gaeta, Mariano Mollica, Maurizia Lanza, Anna Perfetti, Valentina Di Spirito, Giuseppe Fiorentino

**Affiliations:** 1 Department of Respiratory Pathophysiology, Monaldi Hospital, Azienda Ospedaliera dei Colli, 80131 Naples, Italy; anna.annunziata@gmail.com (A.A.); lidia.atripaldi@virgilio.it (L.A.); roberto.rega@ospedalideicolli.it (R.R.); mollicamariano@gmail.com (M.M.); maurizia.lanza85@gmail.com (M.L.); anna.perfetti@ospedalideicolli.it (A.P.); valentina.dispirito@ospedalideicolli.it (V.D.S.); giuseppefiorentino1@gmail.com (G.F.); 2 Pneumology Department, Hospital Universitario Severo Ochoa, 28911 Leganés, Madrid, Spain; 3 Faculty of Medicine, Universidad Alfonso X el Sabio, 28691 Villanueva de la Cañada, Madrid, Spain

**Keywords:** Birt–Hogg–Dubé syndrome, *FLCN*, pulmonary cysts, spontaneous pneumothorax, diffusing capacity, renal cell carcinoma, diagnostic delay, rare disease, case series report

## Abstract

**Background/Objectives**: Birt–Hogg–Dubé (BHD) syndrome is a rare autosomal dominant disorder caused by germline pathogenic variants in the folliculin (*FLCN)* gene. Although it carries a substantial lifetime risk of renal cell carcinoma, its earliest manifestations are typically pulmonary cysts and spontaneous pneumothorax, which are frequently misclassified as primary spontaneous pneumothorax, resulting in diagnostic delay and inadequate oncological surveillance. We aimed to characterise the real-world phenotypic spectrum of BHD encountered in a respiratory referral setting. **Methods**: We retrospectively describe seven consecutive patients with genetically confirmed BHD syndrome diagnosed at our tertiary referral centre between 2022 and 2024. Demographic data, smoking history, *FLCN* variants, pneumothorax episodes, high-resolution computed tomography (HRCT) findings, pulmonary function tests and extrapulmonary neoplasms were collected. Reporting followed the PROCESS 2020 guideline. **Results**: Mean age at genetic diagnosis was 53.1 years (range 41–64). All seven patients had multiple thin-walled pulmonary cysts on HRCT, with the typical basal, subpleural and paramediastinal distribution; three had a pneumothorax history. Despite largely preserved spirometry—mean forced expiratory volume in 1 s (FEV1) of 82.4% predicted—the diffusing capacity of the lung for carbon monoxide (DLCO) was reduced in five patients (mean of 67.4% predicted) and was the most frequently affected functional parameter, although the overall functional picture was heterogeneous. Five patients had solid neoplasms (one renal, one colorectal, one thyroid/parathyroid, one ovarian, one lung adenocarcinoma). **Conclusions**: In this referral-based case series, pulmonary cysts were a constant finding and DLCO was the most frequently reduced functional parameter, although the functional picture varied across patients. These descriptive observations are hypothesis-generating and require prospective, controlled validation—including comparison with other diffuse cystic lung diseases—before any diagnostic algorithm can be proposed.

## 1. Introduction

Birt–Hogg–Dubé (BHD) syndrome is a rare autosomal dominant genodermatosis caused by germline inactivating variants in the folliculin (*FLCN*) gene, located on chromosome 17p11.2 and encoding the protein folliculin [1,2].

Folliculin acts as a tumour suppressor; under physiological conditions it associates with the interacting partners FNIP1 and FNIP2 and modulates the AMP-activated protein kinase (AMPK) and downstream mammalian target of rapamycin (mTOR) signalling pathways [3]. Loss of folliculin function dysregulates these pathways and disturbs autophagy, energy metabolism, cell adhesion and proliferation [4], predisposing affected individuals to the hamartomatous and neoplastic lesions that constitute the classical clinical triad of cutaneous, pulmonary and renal manifestations.

Cutaneous manifestations typically appear in the second to third decade of life and include fibrofolliculomas (benign hair follicle tumours considered pathognomonic of BHD), trichodiscomas and acrochordons, predominantly distributed on the face, neck and upper trunk [5].

Cyst formation is thought to derive from impaired cell–cell adhesion in the alveolar epithelium, rendering the parenchyma susceptible to mechanical stress related to respiration (the so-called “stretch hypothesis”) [6]. In contrast with other cystic lung diseases, BHD-related cysts are typically located in the basal, subpleural and paramediastinal regions, often with irregular or lenticular shapes [7]. Throughout this report, the term “atypical cysts” denotes this pattern: multiple, bilateral, thin-walled cysts with a basal, subpleural and paramediastinal predominance, frequently of irregular, ovoid or lenticular morphology and often abutting the proximal pulmonary vasculature—in contrast with the diffuse, uniform, randomly distributed cysts of lymphangioleiomyomatosis and the nodule-associated, bizarrely shaped cysts of pulmonary Langerhans cell histiocytosis.

Pulmonary involvement is highly penetrant: multiple pulmonary cysts are detectable in approximately 80–90% of adult patients [8]. Spontaneous pneumothorax is the most relevant respiratory complication, occurring in 30–40% of patients, and the recurrence rate may exceed 70% because of the persistent parenchymal alteration [9].

The most clinically significant complication of BHD syndrome is the development of renal neoplasms [10]. Approximately 30–35% of patients develop renal cell carcinoma, typically earlier than sporadic disease (often between 40 and 50 years), often bilateral and multifocal, and with a histopathological spectrum dominated by hybrid oncocytic–chromophobe tumours, pure chromophobe carcinomas and oncocytomas [11,12]. Less commonly, more aggressive variants such as clear-cell or papillary carcinoma are observed.

Because pulmonary involvement typically precedes renal neoplasia by years, patients often come to medical attention because of pneumothorax in young adulthood or because of incidental cysts on chest computed tomography (CT).

Despite this oncological risk, BHD syndrome remains markedly under-recognised, with substantial diagnostic delay [13]. These presentations are frequently misclassified as primary spontaneous pneumothorax or, in smokers, as early bullous emphysema [14]. On the basis of the published literature, rather than of outcome data, earlier recognition of an atypical cystic phenotype has been proposed as a means of enabling renal oncological surveillance, surgical pleurodesis when indicated, and cascade screening of at-risk relatives [15].

Building on this rationale, and in keeping with the broader challenges of diagnosing and managing chronic respiratory diseases, we describe a real-world case series of seven consecutive patients with genetically confirmed BHD syndrome diagnosed at our tertiary respiratory centre. Our aim is to characterise the phenotypic spectrum encountered in routine practice and to highlight the diagnostic and management challenges—from the delayed recognition of an atypical cystic phenotype to long-term renal and oncological surveillance—that this rare chronic lung disease poses to the practising pulmonologist.

## 2. Materials and Methods

### 2.1. Study Design and Participants

We conducted a retrospective observational case series of consecutive patients with genetically confirmed BHD syndrome evaluated at the Respiratory Pathophysiology Unit, Monaldi Hospital, Azienda Ospedaliera dei Colli, Naples, Italy, between January 2022 and December 2024. Inclusion criteria required the identification of a germline pathogenic or likely pathogenic variant in the *FLCN* gene, classified according to American College of Medical Genetics and Genomics (ACMG) and Association for Molecular Pathology (AMP) guidelines. All patients provided written informed consent for the collection, analysis and anonymous publication of their clinical, imaging and genetic data. The study was conducted in accordance with the principles of good clinical practice and ethical standards for medical research. Reporting follows the PROCESS 2020 statement for case series studies.

### 2.2. Clinical, Functional and Imaging Assessment

For each patient, we systematically extracted demographic data, smoking history (never/former/current smoker), age at first respiratory symptom, age at genetic diagnosis, history of spontaneous pneumothorax (number of episodes, laterality, management and surgical interventions), dermatological manifestations, and documented extrapulmonary neoplasms from electronic medical records. Two investigators (A.A., G.F.) independently reviewed all clinical data to ensure accuracy and completeness. Pulmonary function testing comprised spirometry, static lung volumes by body plethysmography, and single-breath diffusing capacity for carbon monoxide (DLCO), were performed using the MasterScreen Body System with SentrySuite software, version 2.19 (Jaeger, Hochberg, Germany), according to American Thoracic Society/European Respiratory Society (ATS/ERS) 2019 technical standards [16]. Predicted values were derived from the Global Lung Function Initiative (GLI) 2012 [17] and 2017 [18] reference equations, which were age- and ethnicity-adjusted. DLCO values were corrected for haemoglobin concentration when available. Because of the retrospective design and the change in pulmonary function equipment/software during the inclusion period, LLN and z-score outputs were not uniformly available across the cohort. To ensure consistent reporting across the entire cohort, spirometric indices, lung volumes and DLCO were therefore expressed and interpreted as percentages of predicted values rather than according to LLN/z-score thresholds.

High-resolution computed tomography (HRCT) of the chest was performed using standardised protocols (1.25 mm slice thickness, supine position, full inspiration and expiration phases) and systematically reviewed by a thoracic radiologist for the presence, number, distribution, morphology and size of pulmonary cysts, as well as for pneumothorax or other thoracic abnormalities. HRCT scans were reviewed clinically by a single thoracic radiologist; images were not independently double-read and no quantitative morphometric analysis (automated cyst counting, volumetry or burden scoring) was performed. Extrapulmonary neoplasms were ascertained from documented personal medical history and existing clinical records, not from a prospective, protocolised oncological screening of the cohort; the proportion of neoplasms therefore reflects historical diagnoses in a referral population and is susceptible to ascertainment and selection bias.

### 2.3. Genetic Analysis

Genetic testing for *FLCN* variants was performed at accredited clinical genetics laboratories using peripheral blood DNA. Testing comprised either targeted Sanger sequencing of the *FLCN* gene or inclusion in multi-gene panels for pneumothorax/cystic lung disease, followed by confirmatory Sanger sequencing. Large genomic rearrangements were assessed by multiplex ligation-dependent probe amplification (MLPA) when indicated. All variants are reported according to Human Genome Variation Society (HGVS) nomenclature standards using the NM_144997.7 reference transcript. Pathogenicity was classified according to ACMG/AMP 2015 guidelines; only pathogenic or likely pathogenic variants were included. MLPA for large *FLCN* rearrangements was performed only when sequencing did not identify a causative variant in a patient with a compatible phenotype and was therefore not undertaken in all patients.

**Figure 1:**
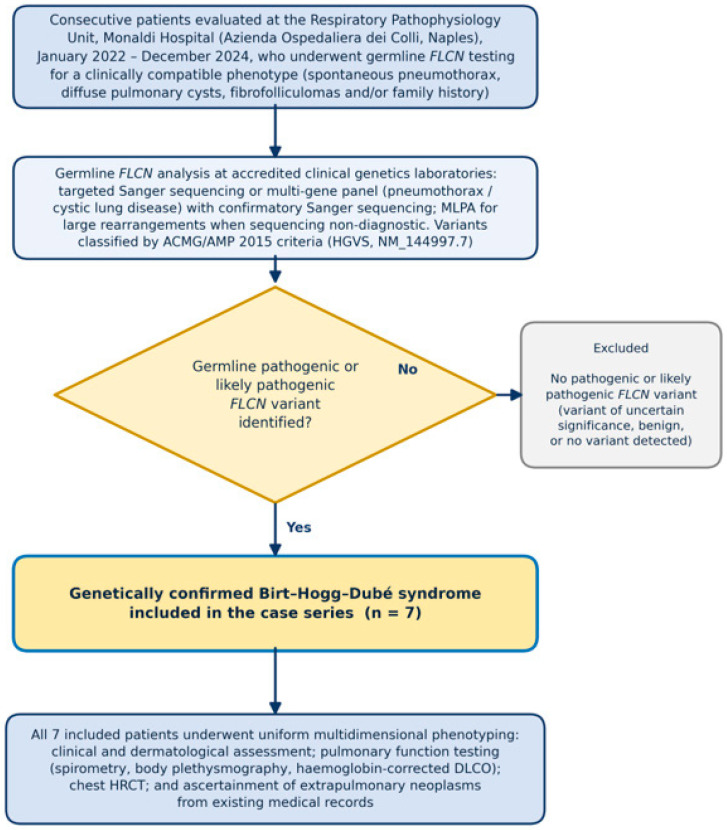
Flow diagram of retrospective patient identification, genetic confirmation, and inclusion in the case series. Consecutive patients evaluated at our centre between January 2022 and December 2024 who had undergone *FLCN* testing were retrospectively assessed. Only patients carrying a pathogenic or likely pathogenic *FLCN* variant according to ACMG/AMP criteria and with complete multidimensional phenotyping were included, yielding the final cohort of seven patients.

Figure 1 illustrates the retrospective screening of consecutive patients who underwent *FLCN* testing, the identification of those carrying pathogenic or likely pathogenic *FLCN* variants according to ACMG/AMP criteria, and the final inclusion of patients with complete multidimensional phenotyping.

### 2.4. Statistical Analysis

Given the small sample size and descriptive nature of the study, only summary statistics are reported. Continuous variables are expressed as mean and range, and categorical variables as absolute frequencies and proportions. No formal statistical testing was performed. All proportions derived from this case series carry wide confidence intervals inherent to small samples and should not be interpreted as population-level estimates. These data are descriptive; no diagnostic test characteristics (sensitivity, specificity or predictive values) can or should be derived from this series. Because GLI reference equations were used, abnormality of diffusing capacity is most appropriately defined by the lower limit of normal (z-score below −1.645); DLCO is therefore reported as percent predicted for description only, and a fixed 70% threshold is not used as a classifier.

## 3. Results

The cohort included seven patients (four women, three men). Detailed clinical and genetic characteristics are summarised in Table 1 and Table 2.

### 3.1. Demographic and Genetic Characteristics

Mean age at genetic confirmation was 53.1 years (range 41–64). Five of seven patients had never smoked; the remaining two (Cases 4 and 5) were current smokers. All patients harboured pathogenic *FLCN* variants (Table 1). Two patients (Cases 2 and 3) carried the same variant (c.1597_1598delCA), confirming familial autosomal dominant transmission within these relatives. Cases 1 and 6 both carried c.1285dup; these two probands were not known to be related, and c.1285dup—a duplication within the C8 mononucleotide tract of *FLCN*—is the most frequently reported recurrent variant in the syndrome, so its independent occurrence in unrelated individuals is expected. A formal pedigree was not reconstructed, as genetic testing and several of the diagnoses had been performed previously at external centres.

### 3.2. Pulmonary Manifestations and Pneumothorax History

**Figure 2:**
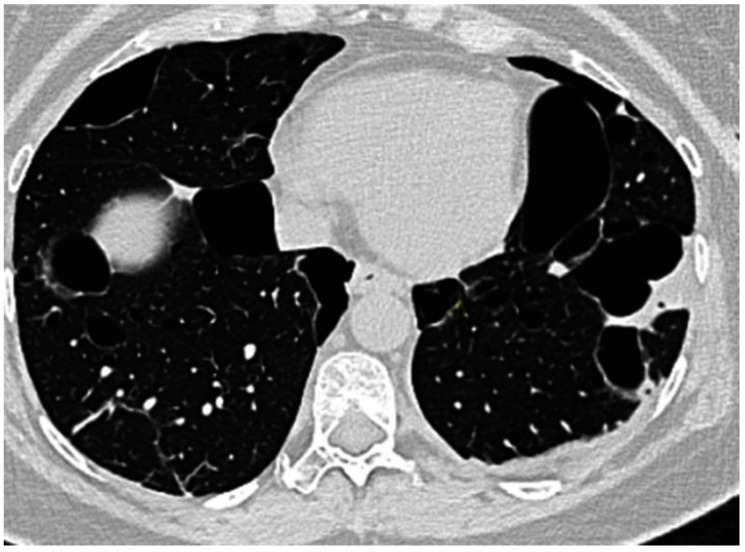
Chest CT, Case 7: bilateral, basally predominant, medial/subpleural thin-walled cysts with paramediastinal involvement.

**Figure 3:**
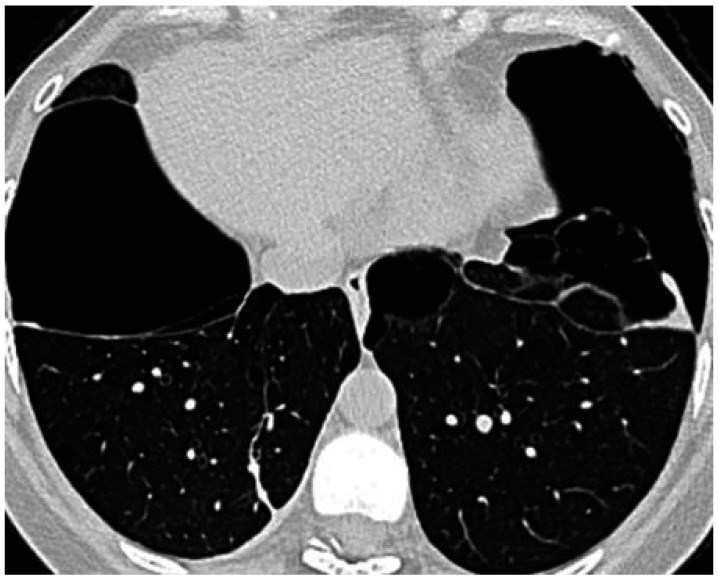
Chest CT, Case 5: left apico-parietal pneumothorax with bilateral cysts of varying shapes and sizes.

**Figure 4:**
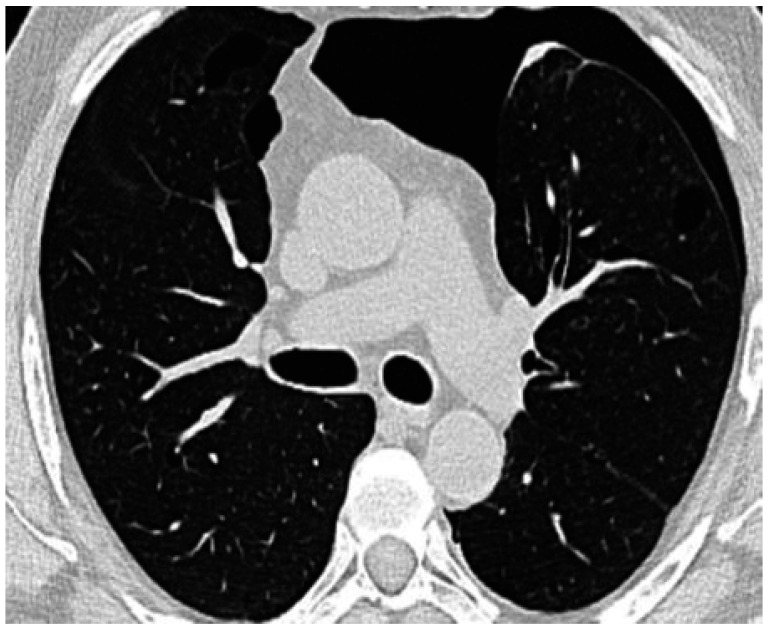
Chest CT, Case 6: bilateral cysts with greater involvement of the lower lung lobes.

Multiple thin-walled pulmonary cysts were present on HRCT in all seven patients, with the typical basal, subpleural and paramediastinal distribution described in BHD (Figure 2, Case 7). Three of seven patients (Cases 2, 4 and 5) had a documented history of at least one episode of spontaneous pneumothorax; Case 2 experienced four recurrent episodes. Case 5 presented with a left apico-parietal pneumothorax (Figure 3). The remaining four patients had no prior pneumothorax at the time of our evaluation, and pulmonary cysts were either incidental findings or detected during work-up for unrelated indications. Two patients underwent thoracic surgery: Case 2 had a left bullectomy with pleurodesis followed by bilateral pleurectomy after recurrent pneumothoraces but continued to experience further pneumothoraces and currently requires ambulatory oxygen during exertion (exertional desaturation was documented on a walking test); Case 6 underwent left bullectomy with subsequent improvement in dyspnoea and reduced air trapping on plethysmography. The bilateral cysts with lower-lobe predominance in this patient are shown in Figure 4.

### 3.3. Pulmonary Function

Baseline spirometry, plethysmography and DLCO data were available for all seven patients (Table 2). The functional pattern was heterogeneous rather than uniform. Ventilatory mechanics were within normal limits in Cases 1, 3 and 5, whereas Cases 4 (FVC 70%, FEV1 60%) and 7 (FVC 69%, FEV1 65%) showed reduced volumes. Air trapping (elevated residual volume and RV/TLC) was present in Cases 2, 4, 6 and 7 (RV 120–135% predicted; RV/TLC up to 51–52%). DLCO was the most frequently reduced functional parameter, with the lowest values clearly below percentages of predicted values; borderline values close to this limit, such as Case 5 at 69% predicted, are best regarded as borderline rather than unequivocally abnormal. The reduction in DLCO was therefore neither uniform nor an isolated finding. Follow-up pulmonary function tests (12–24 months) were available for five patients (Table 2) and showed broadly stable indices, except for Case 6 in whom DLCO declined modestly between baseline and follow-up despite spirometric improvement after surgery. Follow-up testing was not available for Cases 2 and 4, who subsequently elected to continue their surveillance at other centres.

### 3.4. Extrapulmonary Manifestations

Cutaneous lesions consistent with fibrofolliculomas or acrochordons were documented in five of seven patients. Five patients had a personal history of solid neoplasms: one renal tumour (Case 7), one colorectal adenocarcinoma (Case 5), one thyroid/parathyroid lesion (Case 1), one ovarian cancer (Case 6) and one lung adenocarcinoma (Case 4). Although this proportion is higher than that reported in unselected BHD populations, our patients were referred to a tertiary centre, and selection bias toward symptomatic or oncologically complex cases is the most plausible explanation. The detailed histology of the thyroid lesion (Case 1) and of several other neoplasms was not available to the present authors, as these diagnoses had been established previously at external centres; this is reflected in Table 1. Case 4, who had a history of lung adenocarcinoma, was diagnosed and managed outside our institution. Despite review of the available external records, the temporal relationship between prior surgical management of lung adenocarcinoma and pulmonary function testing could not be reliably reconstructed in this retrospective collection. Therefore, it cannot be established with certainty whether the reduced FVC and FEV_1_ observed in this patient reflect the underlying syndrome or may have been influenced, at least in part, by prior surgical management. These values should therefore be interpreted with caution.

## 4. Discussion

This single-centre, real-world case series of seven genetically confirmed BHD patients illustrates several phenotypic features that, although already described in larger international cohorts, deserve renewed emphasis from a clinical–pulmonological perspective. First, pulmonary cysts were universal in our cohort, in agreement with the high penetrance of the radiological phenotype reported in larger series [19]. Second, a history of pneumothorax was present in only three of seven patients, indicating that BHD can present indolently as an incidental cystic phenotype on chest imaging performed for unrelated reasons [9]. Third, pulmonary function was heterogeneous: ventilatory mechanics were preserved in some patients whereas others showed reduced lung volumes or air trapping, and DLCO was the most frequently reduced parameter (reduced, at or below the lower limit of normal, in five of seven patients) rather than a uniform, isolated finding.

Our functional findings are qualitatively consistent with the few available pulmonary function series in BHD, in which DLCO is the most frequently affected parameter while spirometry and total lung capacity remain largely preserved. In the cohort of Holmager et al. (*n* = 101), FEV1/FVC and residual volume were within normal limits and DLCO was only mildly reduced (below 80% predicted in roughly one third of patients; mean approximately 87%), with no significant functional decline over two years, leading those authors to question the need for routine pulmonary function monitoring [20]. Tzilas et al. likewise described the evolution of cystic lung disease in older patients with BHD without consistent functional deterioration [21]. The more pronounced DLCO reduction observed in our series (reduced in five of seven patients, the lowest values lying well below the lower limit of normal) most plausibly reflects the referral nature of our centre and the consequent selection toward more symptomatic or oncologically complex individuals, rather than a generalisable feature of the syndrome; we have tempered our interpretation accordingly.

### 4.1. Radiological Phenotype and Pneumothorax History

In our series, pulmonary involvement was universal, with multiple pulmonary cysts identified on HRCT in all seven patients, consistent with the high penetrance of the radiological phenotype described in larger international cohorts [19]. However, our findings reveal important phenotypic heterogeneity in clinical presentation. Although the literature characteristically associates BHD with recurrent pneumothorax, only three of seven patients in our cohort had a documented pneumothorax history, with Case 2 experiencing four recurrent episodes. The remaining four patients presented with incidental cyst findings during imaging for unrelated indications, suggesting that a substantial proportion of BHD patients may remain asymptomatic from a respiratory perspective despite extensive parenchymal involvement [9].

### 4.2. Age at Diagnosis and Oncological Burden

Genetic confirmation occurred in mid-adult life (mean 53.1 years). We acknowledge that, for a syndrome that is frequently asymptomatic and in which malignancy may never develop, a diagnosis in the fifth or sixth decade is not in itself evidence of clinically meaningful delay; moreover, the interval from first respiratory symptom to genetic diagnosis could not be reliably quantified in this retrospective series. Five of seven patients had developed solid neoplasms by the time of BHD diagnosis, including one renal tumour, one colorectal adenocarcinoma, one thyroid/parathyroid neoplasm, one ovarian cancer and one lung adenocarcinoma. Although this proportion exceeds that reported in unselected BHD populations, it likely reflects referral bias toward a tertiary centre where complex or multimorbid cases concentrate. Nevertheless, our case mix underscores the importance of early pulmonary recognition to enable timely renal screening and multidisciplinary oncological counselling [10,11,12].

Renal surveillance remains the most evidence-based component of BHD follow-up, given that renal cell carcinoma is the most clinically relevant oncological manifestation, often bilateral or multifocal and arising earlier than sporadic disease. Current guidance favours abdominal magnetic resonance imaging over ultrasound because of its higher sensitivity for small renal lesions; surveillance is generally recommended from age 20 onwards and at regular intervals thereafter, with shorter intervals in patients with a family history of renal cancer or with suspicious lesions [22,23].

A baseline dermatological examination is useful to document fibrofolliculomas, trichodiscomas and acrochordons and to support the syndromic diagnosis. Parotid oncocytomas and other parotid neoplasms have also been reported in BHD; thyroid nodules and occasional thyroid carcinomas have been described, but the association is not yet robust enough to justify universal thyroid cancer surveillance [5,22,23].

The role of colorectal surveillance in BHD remains debated. Reports of an association between BHD and colorectal neoplasia have accumulated for decades, but the evidence is heterogeneous; recent data suggest that colorectal risk may be variant-dependent, with certain *FLCN* sequences (e.g., c.1285dupC and c.1177-5_1177-3delCTC) potentially conferring greater risk [24]. A pragmatic, individualised approach therefore seems reasonable: standard population-based colorectal screening for most patients, with earlier colonoscopy in those with a strong family history of colorectal cancer or polyposis, a personal history of adenomas, or a high-risk *FLCN* genotype [22,24].

Lung cancer in BHD is uncommon but has been reported; described lesions range from atypical adenomatous hyperplasia to adenocarcinoma in situ and invasive adenocarcinoma [25,26]. Whether *FLCN* haploinsufficiency contributes to lung tumorigenesis, and whether this contribution is modified by smoking, remains unresolved. Ovarian, endometrial, central nervous system, musculoskeletal, head and neck and haematological malignancies have been reported in BHD patients, but a definite association has not been established [27].

### 4.3. Surgical Treatment Options

Recurrent, bilateral or otherwise clinically significant pneumothorax in BHD is generally managed surgically, with video-assisted thoracoscopic bullectomy and pleurodesis remaining the most established approach [28]. Pleural covering with polyglycolic acid sheets has been proposed as an alternative in patients with diffuse cystic involvement, with the aim of reducing recurrence while limiting pleural adhesions [29]. Pleural adhesions can complicate subsequent thoracic surgery, including lung transplantation; nonetheless, successful bilateral lung transplantation has been reported in BHD even after prior pleurodesis [30]. In our cohort, two patients underwent surgery, with divergent outcomes (Case 2 with persistent recurrences, Case 6 with symptomatic and functional improvement), supporting an individualised approach informed by cyst burden, distribution, pneumothorax history and pulmonary function.

### 4.4. Strengths and Limitations

This study has several strengths. First, all seven patients had genetically confirmed disease, with pathogenic or likely pathogenic *FLCN* variants classified according to ACMG/AMP criteria and reported using standard HGVS nomenclature; no clinically presumptive or unconfirmed cases were included, which strengthens the diagnostic certainty of the cohort. Second, each patient underwent uniform, multidimensional phenotyping that combined clinical history, dermatological assessment, HRCT and a complete set of pulmonary function tests—including static lung volumes by body plethysmography and haemoglobin-corrected DLCO—so that the respiratory phenotype could be characterised more completely than in series relying on spirometry alone. Third, consecutive enrolment over a defined three-year period limited within-window selection by the investigators and provides a representative picture of the patients who actually reach a respiratory referral centre. Fourth, longitudinal pulmonary function data at 12–24 months were available for five of the seven patients, allowing for a preliminary description of functional trajectories rather than a purely cross-sectional snapshot. Fifth, the report adopts a pulmonology-centred, real-world perspective that highlights the sentinel role of the chest physician and adheres to the PROCESS 2020 reporting guideline, enhancing transparency and reproducibility.

Our study also has several limitations. The small number of patients (*n* = 7) precludes any inferential statistical analysis, and the reported proportions carry wide confidence intervals. Patients were recruited at a tertiary referral centre, with the consequent risk of selection bias toward more clinically complex or multimorbid cases; the high proportion of associated neoplasms in our cohort should therefore not be interpreted as a population estimate. HRCT was reviewed clinically rather than rescored prospectively by independent radiologists with quantitative morphometric analysis, and pedigrees of probands were not systematically reconstructed. A further limitation concerns the retrospective harmonisation of pulmonary function data. Owing to differences in pulmonary function reporting outputs across the study period, LLN and z-score values were not uniformly available for all patients. Therefore, spirometric indices, lung volumes and DLCO were reported and interpreted as percentages of predicted values to ensure consistency across the cohort. This may limit direct comparison with studies using LLN- or z-score-based definitions of abnormal pulmonary function. Moreover, follow-up of pulmonary function and oncological surveillance is short and incomplete, particularly for patients managed surgically. As an additional limitation, in Case 4, the temporal relationship between prior surgical management of lung adenocarcinoma and pulmonary function testing could not be reliably reconstructed; therefore, the reduced FVC and FEV_1_ should be interpreted with caution. Finally, the age at first respiratory symptom was inconsistently recorded, precluding reliable quantification of the interval from first symptom to genetic diagnosis. As an uncontrolled case series, this study cannot establish the diagnostic value of any single functional feature; prospective studies with appropriate comparator cystic lung diseases (e.g., lymphangioleiomyomatosis and pulmonary Langerhans cell histiocytosis) are required. Accordingly, the proportion of associated neoplasms and the magnitude of DLCO reduction reported here should be regarded as descriptive observations specific to a referral population, and not as generalisable estimates of the BHD pulmonary or oncological phenotype.

## 5. Conclusions

In this real-world case series of seven patients with genetically confirmed BHD, pulmonary cysts were common, pneumothorax was variable, and pulmonary function was preserved in some cases while in others it showed reduced lung volumes or air trapping, with DLCO being the most frequently reduced parameter. The recognition of these patients only in mid-adult life supports the role of the pulmonologist as a sentinel clinician for BHD: an atypical cystic phenotype, especially with fibrofolliculomas or acrochordons, should prompt early *FLCN* testing, regardless of smoking history, to allow for early renal surveillance and an individualised assessment of the need for further oncological investigation, guided by the patient’s personal and family history, as well as cascade screening of family members. These observations are descriptive and hypothesis-generating; prospective, controlled studies—including comparison with other diffuse cystic lung diseases—are required before any diagnostic recommendation can be made.

## Figures and Tables

**Table 1:** Demographic, genetic and clinical characteristics of the seven patients with BHD syndrome.

Patient	Sex	Age (Yrs)	Smoking	*FLCN* Variant	Cysts	PTX (*n*)	Skin	Renal	Other Neoplasms
Case 1	F	57	Never	c.1285dup p.(His429Profs*27)	Yes	0	No	No	Thyroid lesion *
Case 2	F	48	Never	c.1597_1598delCA p.(Gln533Glufs *)	Yes	4	Yes	No	None
Case 3	M	55	Never	c.1597_1598delCA p.(Gln533Glufs *)	Yes	0	No	No	None
Case 4	M	54	Current	c.352_353del p.(Phe118Glnfs*14)	Yes	1	Yes	No	Lung adenoca.
Case 5	M	64	Current	c.1285del p.(His429Thrfs*39)	Yes	1	Yes	No	Colorectal ca.
Case 6	F	41	Never	c.1285dup p.(His429Profs*27)	Yes	0	Yes	No	Ovarian ca.
Case 7	F	53	Never	c.1222C > T p.(Gln408 *)	Yes	0	Yes	Yes	None

Variant nomenclature is reported according to HGVS recommendations (reference transcript NM_144997.7); patient-level ACMG classification was pathogenic or likely pathogenic for all variants. PTX, pneumothorax. * The detailed histology of this lesion was not available to the authors, as the diagnosis had been established previously at another centre.

**Table 2:** Pulmonary function tests at baseline (T0) and at follow-up (T1).

Patient	Time	FVC (%)	FEV1 (%)	FEV1/FVC	TLC (%)	RV (%)	RV/TLC (%)	DLCO (%)
Case 1	T0	119	115	82	95	88	22	81
Case 1	T1	118	114	81	95	90	26	80
Case 2	T0	82	76	82	78	135	51	42
Case 2	T1	—	—	—	—	—	—	—
Case 3	T0	107	102	75	90	73	26	95
Case 3	T1	107	105	78	92	101	35	88
Case 4	T0	70	60	72	75	120	45	55
Case 4	T1	—	—	—	—	—	—	—
Case 5	T0	80	84	81	80	97	42	69
Case 5	T1	78	80	79	78	100	43	65
Case 6	T0	90	75	71	104	133	41	64
Case 6	T1	95	90	82	95	104	36	54
Case 7	T0	69	65	81	90	132	52	66
Case 7	T1	72	68	81	97	146	55	64

Values are expressed as percent predicted (% pred) using GLI reference equations. Follow-up testing was unavailable for Cases 2 and 4. DLCO, diffusing capacity of the lung for carbon monoxide; FEV1, forced expiratory volume in 1 s; FVC, forced vital capacity; RV, residual volume; TLC, total lung capacity.

## Data Availability

The de-identified datasets supporting the findings of this study are available from the corresponding author upon reasonable request, subject to applicable ethical and privacy restrictions.

## Abbreviations

The following abbreviations are used in this manuscript:

ACMG	American College of Medical Genetics and Genomics
AMP	Association for Molecular Pathology
AMPK	AMP-activated protein kinase
ATS/ERS	American Thoracic Society/European Respiratory Society
BHD	Birt–Hogg–Dubé
CT	Computed tomography
DLCO	Diffusing capacity of the lung for carbon monoxide
FEV1	Forced expiratory volume in 1 s
*FLCN*	Folliculin gene
FVC	Forced vital capacity
GLI	Global Lung Function Initiative
HGVS	Human Genome Variation Society
HRCT	High-resolution computed tomography
MLPA	Multiplex ligation-dependent probe amplification
mTOR	Mammalian target of rapamycin
PTX	Pneumothorax
RV	Residual volume
TLC	Total lung capacity
